# Working With Community Partners to Implement and Evaluate the Chicago Park District’s 100% Healthier Snack Vending Initiative

**DOI:** 10.5888/pcd11.140141

**Published:** 2014-08-07

**Authors:** Maryann Mason, Hatidza Zaganjor, Christine T. Bozlak, Colleen Lammel-Harmon, Lucy Gomez-Feliciano, Adam B. Becker

**Affiliations:** Author Affiliations: Hatidza Zaganjor, Lucy Gomez-Feliciano, Logan Square Neighborhood Association, Chicago, Illinois; Christine T. Bozlak, University at Albany School of Public Health, Rensselaer, New York; Colleen Lammel-Harmon, Chicago Park District, Chicago, Illinois; Adam B. Becker, Consortium to Lower Obesity in Chicago Children, Ann and Robert H. Lurie Children’s Hospital of Chicago. Dr Mason is also affiliated with the Consortium to Lower Obesity in Chicago Children.

## Abstract

**Background:**

The objective of this case study was to evaluate the acceptability, sales impact, and implementation barriers for the Chicago Park District’s 100% Healthier Snack Vending Initiative to strengthen and support future healthful vending efforts.

**Community Context:**

The Chicago Park District is the largest municipal park system in the United States, serving almost 200,000 children annually through after-school and summer programs. Chicago is one of the first US cities to improve park food environments through more healthful snack vending.

**Methods:**

A community-based participatory evaluation engaged community and academic partners, who shared in all aspects of the research. From spring 2011 to fall 2012, we collected data through observation, surveys, and interviews on staff and patron acceptance of snack vending items, purchasing behaviors, and machine operations at a sample of 10 Chicago parks. A new snack vending contract included nutrition standards for serving sizes, calories, sugar, fat, and sodium for all items. Fifteen months of snack vending sales data were collected from all 98 snack vending machines in park field houses.

**Outcomes:**

Staff (100%) and patrons (88%) reacted positively to the initiative. Average monthly per-machine sales increased during 15 months ($84 to $371). Vendor compliance issues included stocking noncompliant items and delayed restocking.

**Interpretation:**

The initiative resulted in improved park food environments. Diverse partner engagement, participatory evaluation, and early attention to compliance can be important supports for healthful vending initiatives. Consumer acceptance and increasing revenues can help to counter fears of revenue loss that can pose barriers to adoption.

## Background

Interventions in various food environments have been conducted to promote healthful eating. Such interventions include expanding farmers markets (1), increasing fresh food inventory in small stores (2,3), financing programs to support full-service groceries in underserved communities (4), and initiating healthy vending machine programs (5–7). Most healthful food interventions focus on school and community settings. Few have focused on parks (8,9).

Parks are an important public space for promoting health, especially in urban settings with limited open space (10), not only through physical activity but also through access to healthful food. Parks are the second largest public provider of food to children in the United States, serving 985,000 meals in 2011 (11). They can play an important role in improving access to healthful food and beverages. As demand grows for diverse approaches to improving food environments, communities may benefit from information about healthful food initiatives in local, state, and national parks. 

The objective of this case study was to evaluate the acceptability, sales impact, and implementation barriers for the Chicago Park District’s 100% Healthier Snack Vending Initiative, launched in 2011. The central evaluation questions were 1) what were patron and staff reactions to the more healthful snack vending items, 2) how did more healthful snack vending sales change during the initiative, and 3) what barriers need to be overcome to strengthen the initiative?

## Community Context

Chicago has approximately 2.7 million residents, of whom approximately 40% are white, 33% are black, and 29% are Hispanic (12). One in 5 (20.0%) children entering kindergarten and nearly 1 in 3 children (29.2%) entering 6th grade is obese (13).

The Chicago Park District is the largest municipal park system in the United States, with 580 parks and 260 field houses covering more than 8,100 acres. Nearly three-quarters of the district’s programs serve children and youth. In 2012, 188,422 (76%) of the 246,548 program participants were children and youth. Parks are located throughout the city, including in community areas where access to healthful food is limited (14). Chicago is one of the first US cities to improve the food environment of parks through a more healthful snack vending initiative. In April 2010, the district sought bids for a snack vending contract that included nutrition standards for snack items that limited serving sizes, calories, sugar, fat, and sodium for all items. Beverage vending was handled through a separate contract set to expire at a later date and was not included in this initiative.

In August 2010, the district executed a 5-year contract with a large national vendor. The contract called for placing 98 snack vending machines in indoor field houses throughout the park system. No park has more than 1 snack vending machine. The contract states that 100% of items sold will meet the following nutrition standards:

1. No more than 250 kcal per serving;
2. No more than 42 g of added sweetener per 20 oz;
3. No more than 35% of kcal from fat (with the exception of seeds and nuts);
4. No more than 10% of kcal from saturated fat;
5. No trans fats;
6. No more than 35% total weight from sugar and caloric sweeteners (natural fruit juice allowed);
7. No more than 400 mg of sodium per serving;
8. At least 5 items must contain less than 250 mg of sodium per serving;
9. No more than 2 servings per package.

A park district staff member, who is a registered dietitian, led the development of nutrition standards, which are based on guidelines from the Alliance for a Healthier Generation (AHG) and the American Heart Association (AHA). The contract also states that all vending machine items will be priced uniformly ($1 per item at the time of evaluation) to eliminate price as a driver of consumer choice. Except for packages of 100-calorie items, the nutrition content of items was not visible to consumers. Snack items chosen on the basis of the new standards included fruit snacks, granola bars, and baked chips.

The previous snack vending contract, which had no nutrition or pricing requirements, expired 2 years before the initiative and allowed items such as cookies, candies, and chips. These vending machines were removed, providing an opportunity to start fresh with new machines and new items. Before implementation of the initiative, district staff from 4 parks participated in a pilot nutrition training and taste-testing session of new snack items. These sessions were poorly attended and resulted in minimal changes in nutrition knowledge among participants. No communications were made to park patrons about the initiative before installation of the new vending machines. To establish an evidence base for informing future vending contracts, district staff and partners decided to conduct an evaluation of the initiative.

## Methods

### Building community partnerships

The initiative was supported by Chicago’s Healthy Kids, Healthy Communities (HKHC) project, funded by the Robert Wood Johnson Foundation. HKHC supported policy, systems, and environmental changes to improve nutrition and increase physical activity for children outside of school settings. The Chicago HKHC leadership team, composed of staff from 1 lead and 4 supporting community-based organizations (CBOs), a local childhood obesity prevention consortium, and the district, played key roles in supporting the initiative and its evaluation. The CBOs provided a consumer perspective, helped district leadership understand the value of the initiative for park patrons, and helped shape evaluation questions and approaches. District staff led the initiative in the park system and ensured access to district facilities and sales data. The obesity prevention consortium provided content expertise and introductions to national consultants for contract development, and its director of evaluation and community research led the evaluation. The project was approved by the institutional review board at the Ann and Robert H. Lurie Children’s Hospital of Chicago.

### Data collection

The evaluation used quantitative and qualitative research methods. Observation and interview data were collected from a convenience sample of 10 parks representative of the sociodemographic characteristics of Chicago, the variation in park amenities throughout the district, and the geographic regions of the district. Within each region, parks were selected according to the race and ethnicity of surrounding neighborhoods, including 4 predominantly white, 3 predominantly black, 2 predominantly Hispanic, and 1 racially and ethnically mixed neighborhood. Selected parks included a mix of larger regional and smaller neighborhood parks with diversity of space and amenities (Table).

**Table:** Characteristics of Chicago Park District Parks Included in Evaluation of Chicago Park District’s 100% Healthier Snack Vending Initiative

Park	Race/Ethnicity of Surrounding Community	Average Annual Household Earnings in Surrounding Community, $	Geographic Region of City	Wellness Center^a^	Fitness Center^b^	Playground	Fields	Swimming Pool	Other	After School Program Park Kids
1	Black	50,110	South	No	No	Yes	Soccer, football, baseball	No	Basketball, tennis	Yes
2	Black	32,388	South	No	No	Yes	No	Outdoor	Basketball, gym, tennis	No
3	Hispanic	52,060	North	No	No	Yes	Soccer, football, baseball	Outdoor	Basketball, gym, tennis, track, outdoor ice rink, lagoon, pond	No
4	Hispanic and white	45,240	Central	No	No	Yes	Soccer, football, baseball	Outdoor	Basketball, fishing, gymnastic center, tennis, water playground, lagoon, pond	No
5	White	47,934	North	Yes	Yes	No	No	No	Gymnastic center, gym, culinary center, climbing wall, dark rooms	No
6	Hispanic	44,059	North	No	Yes	Yes	Soccer, football, baseball	Indoor and outdoor	Basketball, gyms, skate park, tennis, water playground, volleyball	No
7	Black	42,680	Central	No	Yes	No	Soccer, football, baseball	Outdoor	Basketball, boxing, gym	Yes
8	White	50,821	North	No	Yes	Yes	Soccer, football, baseball	Outdoor	Gym, tennis, theaters	Yes
9	White	61,285	North	No	No	Yes	Baseball	Outdoor	Basketball, fishing, gym, tennis, roller hockey rink, lagoon, pond, wetlands	Yes
10	White	58,182	Central	Yes	Yes	Yes	Soccer, football, baseball	Outdoor	Basketball, beaches, gyms, trails, tennis, lagoon, pond, wetlands	Yes

a Wellness centers provide multifaceted, year-round nutrition and fitness programs. Fitness classes, fitness arcading, and interactive fitness equipment are designed to help children and adults have fun while they get fit. There are 6 wellness centers in the Chicago Park District.

b Fitness centers are fee-based and feature state-of-the-art equipment such as computerized treadmills, cross trainers, upright bikes, recumbent bikes, free weights and benches, cable crossovers, multistation weight machines, and core-focused weight equipment. There are 70 fitness centers in the Chicago Park District.

Research assistants conducted semistructured interviews with park staff in summer 2012. One staff member from each park was selected from a list of volunteers. Staff interviews lasted 20 to 60 minutes and explored staff attitudes toward more healthful health snack vending, snack vending purchasing behaviors, observations of snack vending machine issues (stocking and functioning), and interactions with park patrons and their snack choices. Park staff provided written consent for interview participation.

Research assistants observed consumer snack vending purchases during spring and summer 2012. They conducted observations at each park on different days of the week and times of day to capture data on the diversity of program participation and purchasing behaviors. Research assistants visited each park an average of 2.8 times and recorded items purchased, sex and age of the purchaser (child, teen, young adult, adult), whether the purchaser was alone or with others (categorized as children, teens, young adults, or adults), when the items were consumed (upon purchase or at some later time, unseen by the observer), and by whom (by purchaser or by another). Research assistants stood nearby each vending machine to conduct observations but recorded their observations once patrons left the vicinity to reduce patron awareness of being observed. No consent was obtained for vending purchase observations.

Research assistants observed machine conditions and item compliance and stocking during each park visit from fall 2011 through summer 2012. They examined the number of empty slots in each machine to understand how well machines were stocked. A slot is a spot in a vending machine from which a snack is selected; each machine has 40 slots. District management-level staff also observed conditions, compliance, and stocking during park visits.

Research assistants administered a 16-item survey (in English) with park patrons aged 18 or older during summer 2011 and summer 2012. At each park visit, research assistants approached each patron who was in close proximity to a vending machine. Research assistants explained the survey purpose, invited the patron to participate, and determined their age eligibility. Surveys lasted approximately 2 minutes per patron. Questions assessed perceptions of the new snacks and solicited suggestions for improvements. Support for a healthful beverage initiative was also assessed (but not evaluated as part of this study). Park patrons gave verbal consent for survey participation. No incentives were provided for survey completion.

Monthly sales data for June 2011 through August 2012 were provided by the vendor via electronic files to the district and then transmitted to evaluators. Sales data were provided for 98 vending machines. These data were analyzed by vending machine, by item, and by park location.

### Analysis

Quantitative data were analyzed using SPSS 16.0 (IBM Corporation). Monthly vending sales were analyzed from when the vending machines were first installed (June 211) through the end of the evaluation period (August 2012). Baseline sales data and nutritional contents of vended items from before the initiative were unavailable for comparison because of the 2-year gap in vending services. Research assistants individually analyzed qualitative interview data for recurring themes. They then compared their individual analyses and refined the themes for coding to enhance inter-rater reliability. Refined themes were shared with the evaluation partners for discussion and revision. A manageable number of interview themes made computer software unnecessary for coding and data organization.

## Outcomes

Nine park staff members (1 per park) were interviewed from 9 of the 10 sampled parks. At 10 sampled parks, 130 park patron surveys and 27 patron purchasing observation sessions were completed. Observations of machine conditions and compliance were conducted during 27 purchasing observation sessions and 26 staff visits. Fifteen months of vending machine sales data were analyzed. Twenty-six unique observations of snack vending machine conditions and stocking were completed.

Patrons overwhelmingly approved of the more healthful snack vending items; 88% of those surveyed reported liking the snack vending items they tried. Almost all (98%) patrons purchasing snacks from the vending machines indicated that they would purchase the snacks again. The main reason given for disliking the more healthful snacks was that they were not healthful enough. Almost all interviewed park staff (89%) had tried items from the machines. Of those, 100% reported liking the products they had tried.

Eighty-one purchases were observed during 27 observation sessions: children were involved in 44 purchases, 22 of which were made alone and 22 of which were made with an adult. Teenagers made 18 purchases. Approximately 70% of the snack items were consumed in view of the observer.

**Figure 1:**
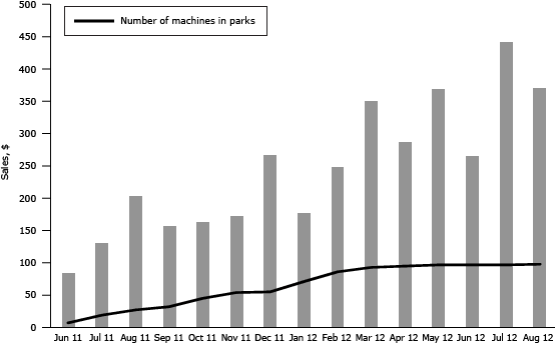
Average monthly sales per machine during the machine deployment period (June 2011 through August 2012). **Table:** 

Generally, sales trends followed the machine deployment schedule with the exception of large dips in January and June 2012 (Figure 1). Overall monthly average sales per machine increased from $84 in June 2011 to $371 in August 2012.

Month and Year	Sales by Month per Machine, $	Number of Machines in Place
June 2011	84	7
July 2011	131	19
August 2011	204	27
September 2011	157	32
October-2011	163	45
November 2011	173	54
December 2011	268	55
January 2012	177	71
February 2012	249	86
March 2012	351	93
April 2012	288	95
May 2012	370	97
June 2012	266	97
July 2012	443	97
August 2012	371	98

Sales exceeded the expectations of both district staff and vendors. Average monthly sales volume per machine also exceeded industry sales estimates of $300 per month for snack vending machines located in “average” locations, which typically have 10 sales per day (15).

Compliance and operational issues during the first 2 years of the contract included stocking of noncompliant items, failure to restock on a timely basis, and machine malfunctions, the latter 2 of which are not specific to stocking of healthful items.

From January through September 2012, 54 instances (or about 0.8% of slots) of stocking noncompliant items were documented. Driver error (stocking of noncompliant items) and mislabeling of items (noncompliant items labeled as compliant in the warehouse) were identified as the primary reasons for noncompliance. To address these issues, the vendor provided drivers with training on the nutrition standards, began pre-boxing compliant items to eliminate the need for drivers to select compliant items from the list of available items, and monitored labeling more closely.

**Figure 2:**
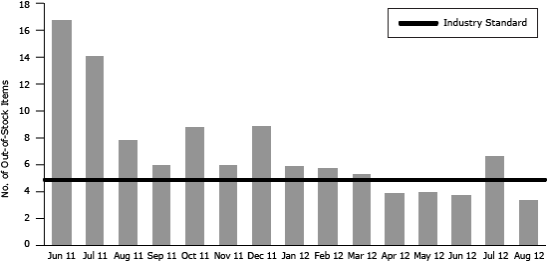
The average number of out-of-stock items (empty slots) per machine at time of refill by month (June 2011 through August 2012). **Table:** 

Restocking of machines was uneven among parks. Some parks reported no problems, while others reported repeated instances of out-of-stock items. The number of slots empty per machine ranged from 0 (a completely stocked machine) to 21 (more than half of slots empty). On average among all observations, 7 slots per machine were empty (Figure 2). In 11 of 15 months, the number of empty slots surpassed the industry average of 5 empty slots per machine.

Month	No. of Out-of-Stock Items per Machine	Industry Standard
June 2011	17.0	5
July 2011	14.3	5
August 2011	8.0	5
September 2011	6.1	5
October-2011	8.9	5
November 2011	6.1	5
December 2011	9.0	5
January 2012	6.0	5
February 2012	5.9	5
March 2012	5.4	5
April 2012	4.0	5
May 2012	4.1	5
June 2012	3.8	5
July 2012	6.8	5
August 2012	3.5	5

Machine malfunctions included failure to vend products, accept money, and give change. More than half (55%) of the malfunctions were failure to vend products and accept money.

## Interpretation

Our evaluation, conducted in a subset of 10 parks, found sufficient evidence of success. The initiative was well received: nearly all patrons and staff reported liking the more healthful offerings. The initiative was also fiscally successful: monthly sales grew over time, surpassing initial projections and industry averages. These findings can inform efforts in other municipalities to improve the nutritional value of snacks offered in park vending machines.

Partnerships played a key role in the success of implementation and evaluation. The joint effort of district staff, community advocates, and policy and obesity experts enabled the creation and execution of the new contract. The partnership enabled the research team to collect data from park patrons and staff, access sales data, and identify implementation issues.

The district worked with the vendor to resolve compliance issues; these issues may have affected sales and are important for entities to consider when examining more healthful vending options. Data on compliance, out-of-stock items, and machine malfunctions were helpful in interpreting sales outcomes and identifying corrective actions. As healthful vending programs become more common, vendors may learn from strategies implemented in our initiative, including the trainer of drivers and the pre-boxing of compliant items.

Even with compliance and stocking challenges, we found that per-machine monthly sales of more healthful vending items increased greatly. One possible explanation for this large increase may have been the absence of vending machines for 2 years before the initiative, which may have caused patrons to fall out of the habit of purchasing snacks at parks. The rise in sales may have occurred as consumer awareness of the new machines increased. The initiative received positive media attention locally, and obesity was gaining more local attention as a critical health issue, which may also have encouraged consumers to buy the new items.

The Centers for Disease Control and Prevention recommends that communities increase availability of more healthful food and beverage choices in public service venues (16). The Chicago Park District’s 100% Healthier Snack Vending Initiative does this by applying nutrition standards consistent with AHA and AHG guidelines. Such approaches can provide opportunities, cues, and support for more healthful behaviors and may be more sustainable than traditional public health approaches focused on individual behavior change (17). Improving access to more healthful foods through machine-vended snacks is a strategy that has demonstrated success in schools and worksites. This strategy can be pursued by using various tactics individually or in combination, including pricing more healthful foods at lower cost, preferential placement of more healthful items in vending machines, signage promoting more healthful foods, increasing the number of more healthful items stocked as a proportion of all items in vending machines, and nutrition education (18–20).

We found no other studies of snack vending interventions that included only items meeting nutritional standards and uniform pricing, so our initiative is unique. This community case study contributes to the emerging evidence that such an initiative can be accepted by consumers and can meet or surpass sales expectations. These are important findings given that fear of revenue loss is often cited as a barrier to implementing healthful vending initiatives. Our experience can help to assuage those fears in other communities and provide support for the district’s new healthful beverage vending initiative. Although we could not compare pre-initiative sales with sales during the initiative, our study found that the average sales per machine increased monthly after the initiative was launched and that sales levels exceeded projections. We also found that 88% of park patrons surveyed liked the more healthful vending items, and 98% would purchase from the machines again, signaling that future revenue loss is unlikely.

As with any program evaluation, generalizing outcomes beyond the evaluation context should be done with caution. The evaluation was limited by sample size and by data collection methods. A larger, randomized sample of parks may have strengthened outcomes but was not feasible because of the initiative’s implementation schedule. Although pre-initiative data would have helped us to understand the overall impact on revenues, the findings of acceptability and increasing revenues over time that exceeded expectations are important and positive.

The initiative has led to improved food environments in Chicago parks. We found that 54.4% of snack vending purchases in parks were made by or for children, so improvements are likely to have a greater effect on children than on adults. Finally, the success of the initiative paved the way for the district to issue a request for proposals for healthful beverage vending, and a contract was awarded in 2013.
